# Comparison Of Mechanical And Chemical Nanocellulose As Additives To Reinforce Recycled Cardboard

**DOI:** 10.1038/s41598-020-60507-3

**Published:** 2020-03-02

**Authors:** Jose Luis Sanchez-Salvador, Ana Balea, M. Concepción Monte, Carlos Negro, Meaghan Miller, James Olson, Angeles Blanco

**Affiliations:** 1 Chemical Engineering and Materials Department, Universidad Complutense de Madrid, Avda. Complutense s/n, 28040 Madrid, Spain; 2 Pulp and Paper Centre, University of British Columbia, 2385 East Mall, V6T 1Z4 Vancouver, BC Canada

**Keywords:** Chemical engineering, Chemical engineering, Nanoparticles

## Abstract

Recycling cycles cause a decrease in mechanical paper properties due to cellulose fiber degradation. The use of cellulose micro/nanofibers (CMF/CNF) to reinforce paper strength has been well studied, although it has been found to have negative effects on drainage. However, the application of CMF/CNF as paper reinforcement is affected by the nanocellulose type. Thus in this study mechanical and chemical treatments in CNF production were compared. Old corrugated container (OCC) pulp used to produce recycled cartonboard was reinforced with 1) CMF from never-dried northern bleached softwood kraft pulp (NBSK) highly refined in a 16-inch low consistency refiner at 1200 rpm and 25 kW of net power; and 2) CNF from NBSK pulp treated by TEMPO-mediated oxidation and homogenization at 600 bars. CMF/CNF and OCC were pulped at the same time and handsheets formed with cationic starch (CS) as retention system. Mechanical, drainage and flocculation properties were evaluated and compared. Data were also compared with other sources of TEMPO CNF. Results show an improvement in mechanical properties, drainage and flocculation when OCC is reinforced with CMF obtained with LCR. Therefore, high fibrillation was not necessary to improve mechanical paper or cardboard properties.

## Introduction

The papermaking industry has achieved an increase of global paper recycling by 12.4 percentage points since 2000 as a result of environmental consciousness^1^. In Europe, the paper recycling rate reached 72.5% in 2017. The collection of paper has increased up to 59.6 million tonnes while paper consumption has increased by 0.5% compared to 2016 to 82.5 million tonnes^2^. However, by increasing the number of recycling cycles of paper and cartonboard, the quality of the fibers gradually decreases due to the chemical and mechanical treatments that occur during paper sheet formation, causing irreversible changes in the fiber structure and properties^3,4^. This hornification of cellulose fibers reduces the bonding potential of recycled fibers with the number of cycles and, consequently, their strength^3,5–7^. Therefore, one of the main challenges in the utilization of secondary fibers is to maintain the quality levels of the products which is continuously increasing following customer demands.

Several strategies have been used to improve mechanical and physical properties of recycled products, such as fiber refining^8,9^, or the addition of strength additives, for instance, cationic starch or synthetic polymers such as polyacrylamides, polyvinylamines or polyvinyl alcohols^10–13^. In the last decade, the addition of cellulose microfibers (CMF) or cellulose nanofibers (CNF) have been explored as potential alternatives as strength agents to reinforce paper and cartonboard due to their ability to form a cross-linked network that improve the strength and rigidity of the paper^14–19^. CMF and CNF are sustainable, renewable and biodegradable with interesting properties such as high specific surface area, high aspect ratio and high mechanical strength^20^.

In general, CMF and CNF are produced by mechanical defibrillation of cellulose fibers, for instance, refining, high-pressure homogenization, microfluidization, grinding, high intensity ultrasonication, etc.^21–24^ Before these treatments, chemical, mechanical, enzymatic or combined pretreatments are used to improve cellulose fibrillation, obtaining individual cellulosic fibers and decreasing energy requirements of mechanical treatments^14,25–31^. Among them, TEMPO-mediated oxidation is the most common pretreatment, producing a high nanofibrillation degree^6,32–34^. Other alternatives such as refining are used as a treatment to produce CMF with lower fibrillation. Cellulose fibers go through a mechanical defibrillation process and are forced to pass inside a refiner, a rotary device equipped with a bar-groove patterned rotor and stator separated by a gap of 0.1–2.5 mm^35,36^. Although CMF and CNF have been successfully obtained from the mentioned technologies, several drawbacks still exist such as high energy consumption and chemical agents required.

Refining has been widely used as pretreatment combined with other defibrillation treatments such as homogenization to obtain CMF/CNF^25,29,30,33,37–40^. Other researchers have studied refining as the main mechanical defibrillation treatment to prepare CMF or CNF without additional treatments or using a low concentration of NaOH in the fibers soak. These CMF/CNF were obtained in high consistency refiners (HCR), where the heterogeneous furnish formed by fibres and fines, water and steam is at a concentration in the range of 10–40%^41,42^; or in low consistency refiners (LCR) at a concentration of 2–6%^26,43^. The pulp suspension acts as an incompressible fluid and is pumped through the refiner using an external pump. At low concentration, the pulp is more homogeneous and, consequently, the refining treatment more uniform than in HCR^26,36^. Among these studies, only some of them use CMF/CNF as an additive to reinforce strength properties in paper or board. In the case of CMF/CNF prepared with HCR, Kumar *et al*.^41^ studied an increase of mechanical properties as tensile and burst indexes in three different papers from hardwood, softwood and bagasse pulps with a CNF addition up to 20%. On the contrary, drainage time is longer with the reinforcement. In the same vein, CNF from LCR with different fines content were used by Johnson *et al*.^26^ to improve paper mechanical and surface properties, although drainage properties were adversely affected. In addition, pretreatments such as enzymatic hydrolysis before LCR defibrillation has been studied by Bharimilla *et al*.^14^ to prepare unbleached kraft paper handsheets reinforced with 5 and 10% of CMF and the mechanical properties were also improved.

However, to our best knowledge there are not any comparison studies about the reinforcement of recycled cartonboard using CMF produced by LCR or CNF obtained by TEMPO oxidation and homogenization. Furthermore, the need of a high degree of nanofibrillation is not clear. Therefore, in this research, nanocelluloses from northern bleached softwood kraft pulp (NBSK) obtained by 1) LCR without any pulp pretreatment; and 2) by TEMPO-mediated oxidation with 5 mmol of NaClO/g of dry pulp and 3 passes in a laboratory homogenizer at 600 bars, were used to reinforce old corrugated containers (OCC). The mechanical (SCT index, tensile index, burst index and porosity), flocculation and drainage properties of the recycled OCC reinforced with CMF or CNF were studied and compared. In addition, the results were compared with published studies that use cartonboard with CNF from other pulp sources obtained by TEMPO-mediated oxidation and homogenized. Additionally, CMF produced by LCR were modified using a pretreatment with NaOH and a post-treatment by homogenization. The effects of both treatments on the final quality of the OCC handsheets have been assessed.

## Materials

OCC was obtained from Räpina Paberivabrik AS (Estonia). Never-dried northern bleached softwood kraft pulp (NBSK) from Canada was used as the cellulose source for the nanocellulose production. Cationic starch (CS) was employed as the retention system in the handsheets. A CS solution was prepared at 6 g/L. 3 g of dry potato CS (Solam, Kristianstad, Sweden) were heated to 90–95 °C for 15 minutes in 200 mL of water under magnetic agitation and covered with aluminum foil. Finally, the CS solution was chilled to ambient temperature and diluted to 500 mL. The CS dose (0.5 g/100 g pulp) was selected based on the optimum doses obtained by Balea *et al*. (2016a) and it was kept the same throughout the study.

## Results and Discussion

### CMF and CNF characterization

**Figure 1:**
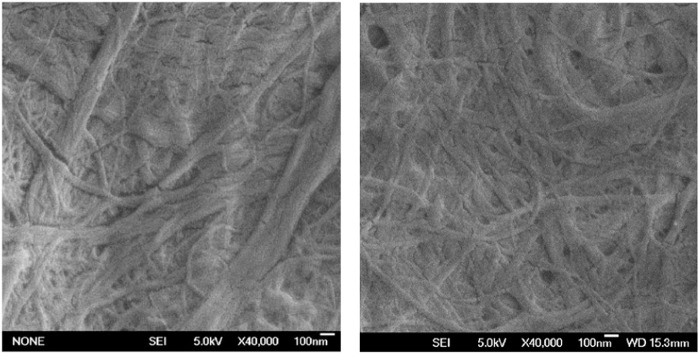
SEM images of LCR-1-CMF and LCR-2-CMF.

CMF produced by LCR (LCR-1-CMF and LCR-2-CMF) at several homogenization passes were characterized as shown in Table 1. The main difference between the CMF samples is the carboxylic group content. LCR-1-CMF has 0.14 mmol COOH/g, while LCR-2-CMF has increased carboxylic groups up to 0.51 mmol COOH/g due to alkali pretreatments of cellulose as NaOH and the presence of heat increased the content of these compounds^44,45^. However, the addition of sodium hydroxide before the LCR does not have influence on other CMF properties without homogenization treatment. In spite of the presence of more carboxylic groups which increases the repulsive forces and helps the defibrillation of cellulose during the mechanical process, the nanofibrillation degree and transmittance indicate that both materials were not effectively nanosized in individual nanofibrils. Cationic demand represents the anionic nature of the fibers and has been traditionally used to determine the extent of fiber delamination of beaten papermaking pulps^46^. In this case, the low cationic demand is expected due to the poor fibrillation and the anionic nature of cellulosic materials suspended in water^40,47^. Figure 1 shows an image of LCR-1-CMF and LCR-2-CMF by SEM in which fibers are visualized and a diameter characterization was done, with a range from 40 nm to 1 µm in both cases. Furthermore, the properties of CMF produced by LCR and with 2 or 4 homogenization passes barely affect the CMF characteristics. Nanofibrillation yield and transmittance measures are quite low and close to CMF without homogenization passes. Charge measurements also are similar to CMF without homogenization. All of that suggests that a homogenization step after LCR does not produce the desired effect, a higher defibrillation of CMF. Therefore, a high refining pretreatment prevents cellulose nanofibrillation by homogenization.

**Table 1:** Characterization of CMF produced by LCR and several homogenization passes at 600 bar.

	LCR-1-CMF			LCR-2-CMF		
Pulp pretreatment	No			NaOH up to pH 9		
Carboxyl groups (mmol COOH/g)	0.14			0.51		
Homogenization passes	0	2	4	0	2	4
Nanofibrillation Yield (%)	5.6	8.9	16.9	4.7	16.2	18.5
Polymerization Degree (No. Monomers)	2165	2100	1975	2120	1675	1565
Cationic Demand (meq/g)	0.041	0.047	0.069	0.064	0.050	0.055
Zeta Potential (mV)	−20.5	−18.1	−23.2	−25.9	−14.0	−18.8
Transmittance 400 nm	1.6	2.2	3.7	0.75	2.5	2.9
Transmittance 800 nm	12.6	15.4	22.0	7.6	18.8	20.7

Results from the characterization of 5T-CNF, produced by TEMPO-mediated oxidation and three homogenization passes, are shown in Table 2. As its name indicates, 5T-CNF have a high nanofibrillation yield, independent of the homogenization passes; while LCR-CMF present low nanofibrillation yield with and without alkaline pretreatment. In addition, high transmittance readings corroborate the high fibrillation of the CNF. On the other hand, 5T-CNF polymerization degree is inferior to LCR-CMF that indicate the break of cellulose chains. As for the carboxyl groups, TEMPO pretreatment increases the carboxyl groups (0.53 mmol COOH/g pulp), with a similar effect as the alkaline pretreatment, and a higher cationic demand and zeta potential.

**Table 2:** Characterization of CNF produced by TEMPO-mediated oxidation and 3 homogenization passes at 600 bar.

Carboxyl groups (mmol COOH/g)	0.53
Nanofibrillation Yield (%)	93.8
Polymerization Degree (No. Monomers)	287
Cationic Demand (meq/g)	0.561
Zeta Potential (mV)	−41
Transmittance 400 nm	79.9
Transmittance 800 nm	89.6

### Effect of CMF/CNF on mechanical properties

#### CMF produced by mechanical treatment (LCR-1-CMF)

**Figure 2:**
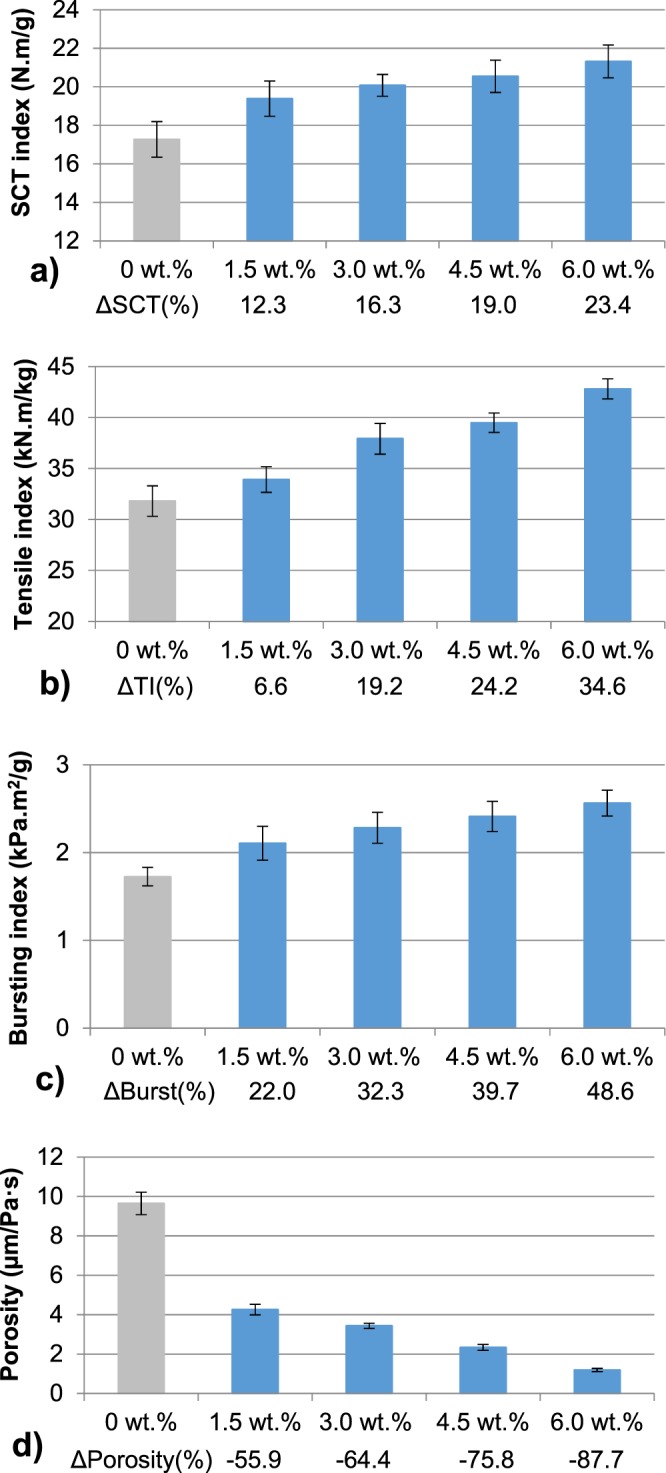
Mechanical properties of OCC handsheets reinforced with different doses of LCR-1-CMF and CS: (**a**) SCT Index; (**b**) Tensile Index; (**c**) Bursting Index; (**d**) Porosity.

First, the effect of LCR-1-CMF on the mechanical properties of recycled OCC handsheets was studied. Figure 2(a–d) shows the mechanical properties of OCC handsheets with several LCR-1-CMF doses and CS as the retention system. A higher dose of LCR-1-CMF increases SCT, tensile and bursting indexes and decreases porosity. In any case, there is an optimal dose at which mechanical properties stabilize. The use of 6 wt.% LCR-1-CMF increases by 23, 35 and 49% the SCT, tensile and bursting index, respectively, compared to handsheets without CMF. These results are in accordance with Johnson *et al*.^26^ who used refined CNF from softwood pulp with several fines doses on bleached northern hardwood pulp. In that work, 5 and 10 wt.% CNF increases tensile index up to 20 and 45%, respectively. In our case, results are quite similar, and the addition of 4.5 and 6 wt.% of LCR-1 increases tensile index by 24 and 35%, respectively.

#### CNF produced by chemical treatment (5T-CNF)

**Figure 3:**
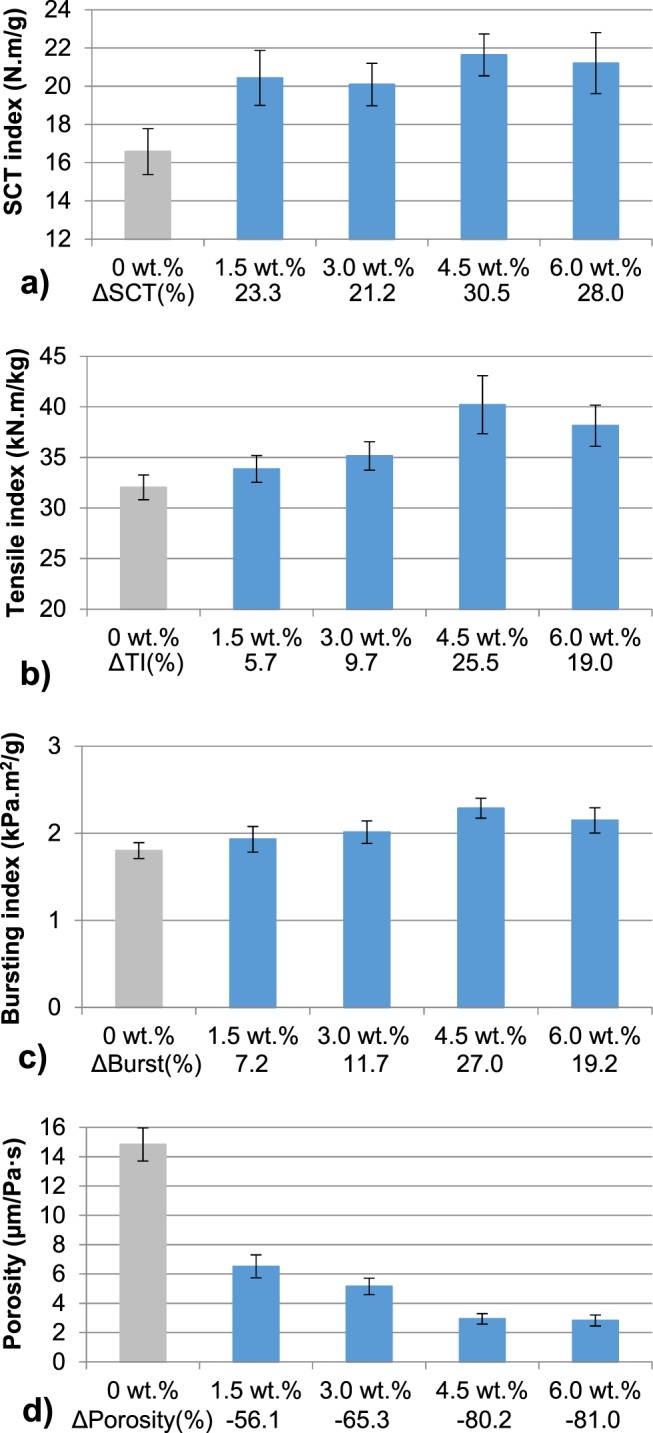
Mechanical properties of OCC handsheets reinforced with different doses of 5T-CMF and CS: (**a**) SCT Index; (**b**) Tensile Index; (**c**) Bursting Index; (**d**) Porosity.

As a comparison, the mechanical properties of recycled OCC handsheets reinforced with 5T-CNF were studied. Figure 3(a–d) shows the mechanical properties of OCC handsheets with several doses of 5T-CNF and CS. An increase of 5T-CNF dose improves the results of tensile and burst index up to 4.5 wt.% of 5T-CNF. This dose is the optimal in which tensile index and burst index increase by 25.5 and 27.0%, respectively. Higher doses of CNF do not improve the mechanical properties and the differences between 4.5 and 6 wt.% dose are negligible. Finally, porosity is also reduced with the incorporation of CNF. 4.5 and 6 wt.% of 5T-CNF reduces porosity by around 80%.

Comparing the mechanical properties of OCC handsheets reinforced with both types of CNF/CMF, OCC handsheets with 5T-CNF subjected to compression forces have better results than LCR-1-CMF. However, tensile index has similar results in both CMF/CNF and improves with the dose up to 4.5wt.%. A higher dose of 6 wt.% LCR-1-CMF improves the tensile index results by about 35% whereas the same dose of 5T-CNF reduces tensile strength. Finally, bursting index is increased by using LCR-1-CMF. This fact is in accordance with El-Hosseiny and Anderson^48^, that indicate an improve of bursting index with tensile strength and with the length of fibers as is the case of LCR-1-CMF.

#### Comparison of CMF/CNF treatments and sources in OCC mechanical properties

Other research has also evaluated the mechanical properties of OCC handsheets reinforced with CMF/CNF produced with different treatments and sources. This is the case of the study of Balea *et al*.^6^ They produced two CNF chemically treated with 2,2,6,6-tetramethylpiperidine-1-oxyl (TEMPO)-mediated oxidation from a never dried refined *Eucalyptus globulus* ECF bleached kraft pulp (TCNF-E) and never dried bleached pine pulp (TCNF-P). 5 mmol of NaClO/g of dry pulp were used in TEMPO pretreatment and once the pulp was oxidized, six passes of homogenization at 600 bar were applied. Table 3 compares those data with our results using LCR-1-CMF and 5T-CNF. The main difference between CMF/CNF treated with TEMPO and LCR is shown in increased transmittance and nanofibrillation yield with TEMPO. This fact indicates TEMPO fibers are nanosized and LCR fibers are mainly microfibers. Other parameters such as polymerization degree in LCR-1-CMF is quite higher than in TEMPO-mediated oxidation CNF, due to mechanical treatments not breaking cellulose chains. As expected, the number of carboxyl groups of CNF chemically treated with TEMPO is higher than those with only mechanical treatments (LCR-1-CMF) but similar to the ones pretreated with NaOH (LCR-2-CMF), as shown in Table 1.

**Table 3:** Comparison of different treatments used to prepare CNF/CMF.

	LCR-1-CMF	5T-CNF	TCNF-P*	TCNF-E*
Carboxyl groups (mmol COOH/g)	0.14	0.53	0.75	0.59
Transmittance 400 nm (%)	1.6	79.9	77.5	83.5
Transmittance 800 nm (%)	12.6	89.6	88.9	94.8
Cationic demand (meq/ g CNF)	0.041	0.561	0.902	1.139
Nanofibrillation Yield (%)	5.6	93.8	>95	>95
Polymerization Degree (No. Monomers)	2165	287	300	440

^*^According to Balea *et al*. (2016a).

Table 4 shows the effects of LCR-1-CMF and 5T-CNF on mechanical properties of OCC compared with the effects of TCNF-E and TCNF-P. In both cases, to evaluate the effect of CMF/CNF, the improvements in mechanical properties were evaluated in relation to OCC without micro/nanofibers and with 0.5 wt.% CS. Tensile Index shows the best results with 6 wt.% LCR-1-CMF (34.6%), although handsheets with TEMPO-mediated oxidation have a similar behavior with a maximum tensile improvement with a dose of 4.5 wt.% and an increase of 25.5, 27.3 and 30.1%, for 5T-CNF, TCNF-P and TCNF-E respectively. A higher dose of TEMPO-CNF decreases the tensile index. As for SCT index, 5T-CNF has better results than the others for all doses. 5T-CNF and TCNF-P, both from softwood pulp and 5 mmol of NaClO/g CNF, have large differences that indicate the variation with the CNF source in spite of using the same type of furnish. However, Bursting Index is higher when CNF/CMF is treated with a mechanical process in which the final length of fibers is greater. Finally, in all cases, porosity decreases in the same way with the addition of the same dose of the four additives.

**Table 4:** Comparison of mechanical properties of OCC handsheets reinforced with CNF with different treatments and doses.

CMF/CNF doses	CMF/CNF treatment	ΔTensile Index (%)	ΔSCT Index (%)	ΔBursting Index (%)	Porosity (µm/Pa·s)
3 wt.%	LCR-1-CMF	19.2	16.3	32.3	3.4
	5T-CNF	9.7	21.2	11.7	5.2
	TCNF-P	14.1	6.5	23.1	3.3
	TCNF-E	14.7	11.6	20.0	3.2
4.5 wt.%	LCR-1-CMF	24.2	19.0	39.7	2.3
	5T-CNF	25.5	30.5	27.0	2.9
	TCNF-P	27.3	15.5	28.5	2.3
	TCNF-E	30.1	18.5	33.9	1.5
6 wt.%	LCR-1-CMF	34.6	23.4	48.6	1.2
	5T-CNF	19.0	28.0	19.2	2.8
	TCNF-P	21.8	19.2	35.9	1.6
	TCNF-E	24.6	18.2	21.9	1.1

### Effect of pulp pretreatment and homogenization on mechanical properties

**Figure 4:**
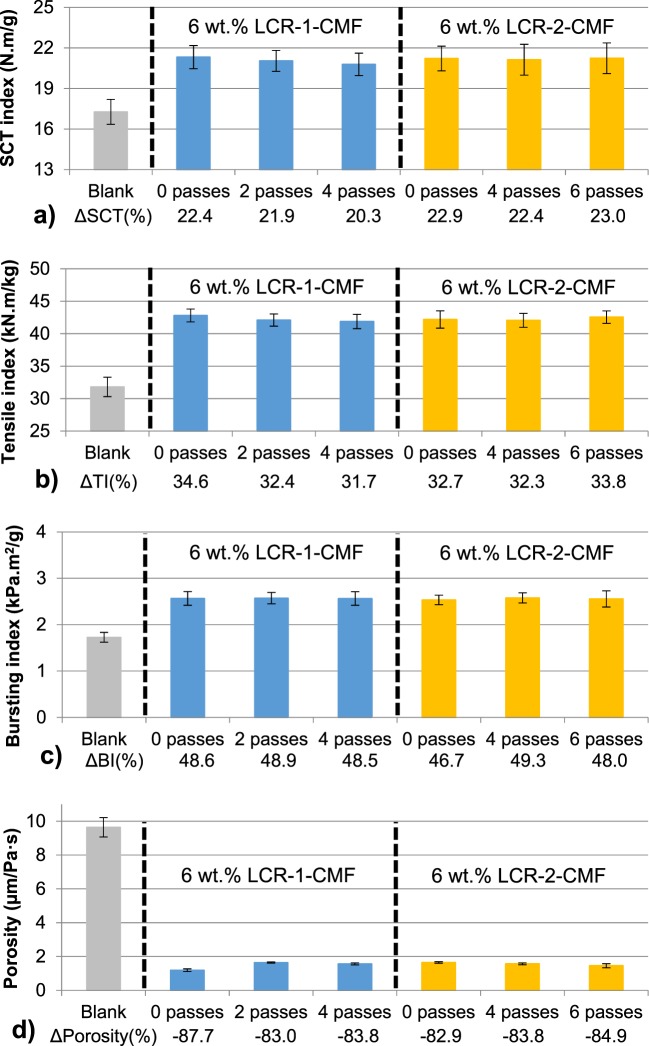
Mechanical properties of OCC handsheets reinforced with LCR and different homogenization passes: (**a**) SCT Index; (**b**) Tensile Index; (**c**) Bursting Index; (**d**) Porosity.

Once the effect of LCR-1-CMF was assessed to reinforce the recycled OCC, the sodium hydroxide pulp treatment prior to LCR was studied to evaluate its influence on mechanical properties of OCC handsheets. In addition, the effect of the homogenization treatment after LCR was also evaluated by adding CS in the same amount as CMF (6 wt.%). Figure 4(a–d) shows SCT index, tensile index, bursting index and porosity, respectively. Results show a similar increase in mechanical properties for both types of LCR-CMF regardless of the number of passes in the homogenizer. The effect of 6 wt.% LCR-1-CMF and LCR-2-CMF was an improvement of SCT index by about 20–23%, increased tensile index of about 31–35% and an increase of 46–49% in bursting index. As for porosity, CNF takes up the pores available and porosity decreases in the same quantity for all experiments. The effect of NaOH pretreatment to modify pH does not affect mechanical properties and OCC reinforced with LCR-2-CMF does not have differences compared to OCC with LCR-1-CMF. Furthermore, the application of homogenization passes is inefficient. In spite of the high energy consumption required, mechanical properties are maintained. This fact is in accordance with Bharimilla *et al*.^14^ who studied the use of CMF produced by enzymatic pretreatment and LCR and the differences with an additional mechanical treatment, by microfluidization. In this case, tensile index, burst and breaking length of CMF with LCR and microfluidization not only did not improve the results of CMF produced by LCR, they made them worse. Overall, a homogenization process or NaOH treatment are unnecessary stages to produce CMF to improve OCC mechanical properties.

### Effect of LCR-1-CMF vs. 5T-CNF on retention and drainage processes

**Figure 5:**
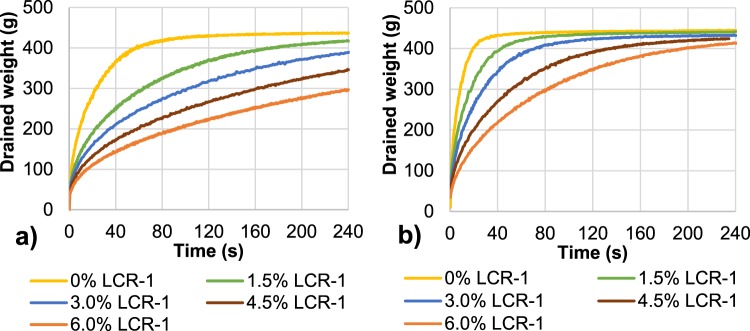
Effect of LCR-1-CMF dose on drainage (**a**) without retention system; (**b**) with 0.5 g CS/100 g pulp as retention system.

**Figure 6:**
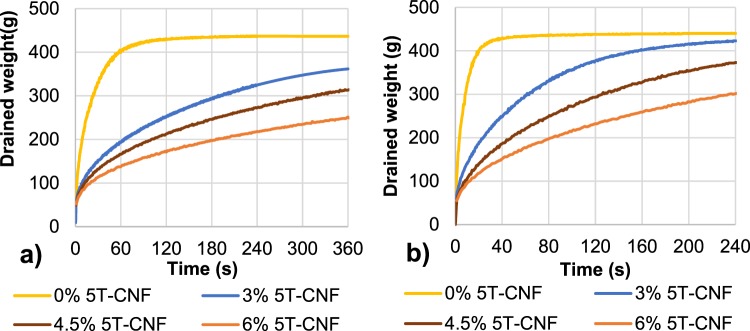
Effect of 5T-CNF dose on drainage (**a**) without retention system; (**b**) with 0.5 g CS/100 g pulp as retention system.

The effect of LCR-1-CMF on retention and drainage of recycled OCC was assessed with and without a retention system. As Fig. 5a shows, a higher dose of LCR-1-CMF retained more water in the pulp, making the drainage process more difficult. However, this effect can be reduced with the incorporation of CS as a retention agent (Fig. 5b). The effects of LCR-1-CMF on OCC drainage were also compared with CNF treated by TEMPO-mediated oxidation, 5T-CNF (Fig. 6). As previously, a higher dose of 5T-CNF requires a higher drainage time, whereas the addition of a retention agent improves drainage. The results obtained are in accordance with Taipale *et al*.^16^, Johnson *et al*.^26^ and Balea *et al*.^40^, that indicate the addition of CMF to a pulp suspension worsens the drainage rate and the addition of retention systems can counteract the effect of CMF.

To easily compare drainage results, drainage time was compared when 300 g of water were drained (W300). W300 was compared with and without CS, and in all cases an increased dose of CMF or CNF increases the retention time, because they block pores between fibers and reduce the water drained^49^.

**Figure 7:**
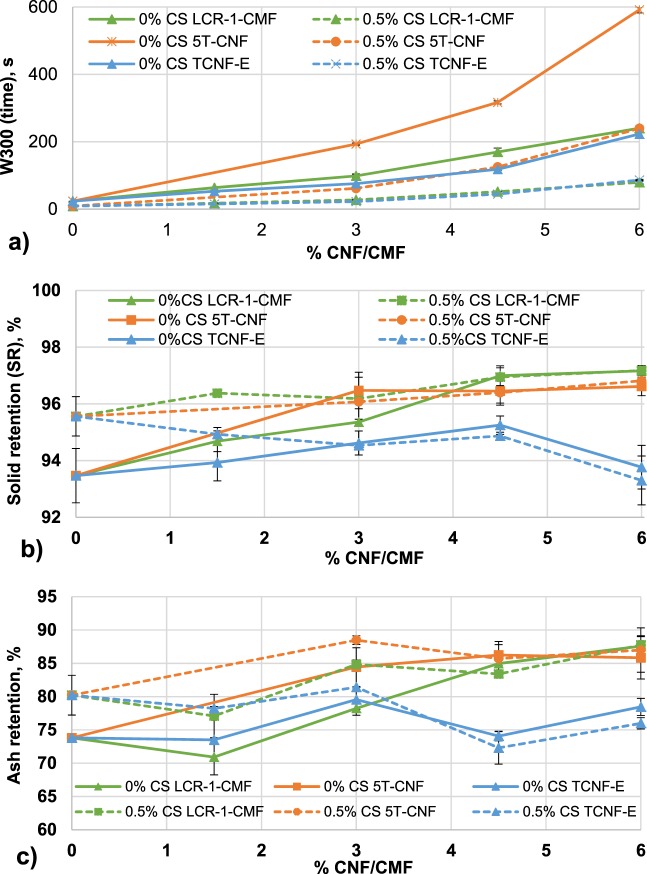
Drainage effect of LCR-1- CMF, 5T-CNF and TCNF-E: (**a**) drainage time (W300); (**b**) solids retention (**c**) ash retention.

In addition, Fig. 7a shows the different drainage behavior of LCR-1-CMF and 5T-CNF with published data using TCNF-E in OCC handsheets. When a retention system is not used, the W300 of the recycled pulp with the same dose of 5T-CNF was higher compared to the addition of LCR-1-CMF and TCNF-E which indicates a worse drainage. Furthermore, when 0.5 wt.% CS was used as a retention system, the retention system effect prevails over the type of CNF/CMF, reducing around 60–70% drainage time for all CNF/CMF doses in the three cases. LCR-1-CMF and TCNF-E maintain better results in drainage with insignificant differences between them.

Figure 7b shows total solids retention of recycled OCC with CNF/CMF, determined through gravimetric analysis. Solids retention without CNF/CMF shows the worst solids retention (93.5 and 95.6%, with 0% and 0.5% CS, respectively). This retention is lower without a retention system. For the three treatments, at low CNF/CMF doses, CS reduces the amount of solids that pass through the drainage membrane. However, the use of 4.5 or 6 wt.% fibers minimizes the effect of the retention system. OCC pulp with CMF/CNF produced from NBSK have the best results reaching with 6 wt.% LCR-1-CMF/5T-CNF the 97 wt.% of solids retained independently the treatment to prepare CNF/CMF from NBSK and the use of retention system. On the other hand, when OCC is reinforced with higher doses of TCNF-E solids retention worsens the results of OCC without micro/nanofibers. A high diameter of fibers as LCR-1-CMF avoid the pass through the membrane while a low diameter range worsens the retention and CS is not capable to retain them.

Finally, ash retention as shown in Fig. 7c has similar results as solids retention due to CMF/CNF sizes. The best retention is shown with a dose of 6 wt.% of LCR-1-CMF and 5T-CNF with an ash retention value of 88 and 90 wt.% in LCR-1-CMF and 5T-CNF, respectively. Both materials were obtained from NBSK, with higher fiber length than Eucalyptus fibers. However, ash retention in OCC with TCNF-E is quite steady, independent of micro/nanofibers content or the retention system addition and worse values than those obtained from NBSK are achieved. The effect of CS is negligible when 4.5 and 6 wt.% of all cellulose additives are added whereas at low CNF/CMF dosages, CS improve ash retention with the same tendency as described in the solids retention.

### Effect of LCR-1-CMF and 5T-CNF on flocculation

**Figure 8:**
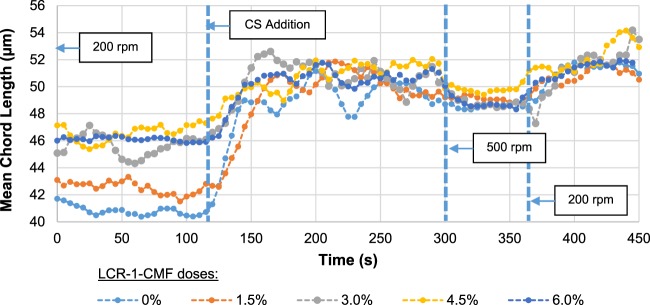
Mean chord Length profile depending on LCR-1 dose.

**Figure 9:**
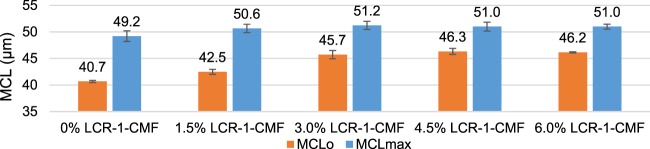
MCL according to LCR-1 dose and CS influence.

The use of micro/nanocelluloses modifies the retention system needs during the flocculation of the pulp flocculation in the wet-end of the paper machine and may alter the retention, drainage and formation processes. Therefore, the flocculation and floc properties of recycled OCC pulp with several LCR-1-CMF and 5T-CNF doses and 0.5% CS were studied. Mean chord length (MCL) during the flocculation process at different LCR-1-CMF doses is shown in Fig. 8. Higher doses in LCR-1-CMF increases the MCL before CS addition (MCLo) until a dose of 3 wt.% LCR-1. From this value, MCLo is stable when the LCR-1 dose increases. On the other hand, after CS addition, MCL has the same maximum value for all CMF dosages and is constant when the stirring is maintained, being stable. The stability in pulp flocculation with CS is in accordance with Merayo *et al*. (2017b), that indicate stable flocs with doses up to 10 mg CS/ g pulp due to hydrogen bonds. When the stirring speed increases to 500 rpm, it was observed that flocs show a high resistance to the high shearing forces due to a small decrease of MCL. Then, stirring speed was decreased again to 200 rpm and flocs were reflocculated achieving the previous MCL value. In the flocculation of OCC without CMF, floc stability is maintained with stirring speed and the incorporation of LCR-1 does not modify this stability. Figure 9 shows the MCLo (prior to CS addition) and the maximum MCL (MCLmax) after CS addition. MCLo increases with LCR-1 dose. At 3 wt.% LCR-1-CMF, MCLo is constant while the MCLmax does not vary with the CMF dose. In general, when the retention system is added, the CNF effect is negligible because the CS reduces the effect of CMF.

**Figure 10:**
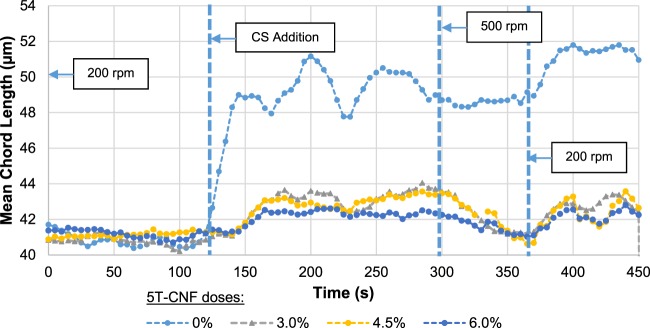
Mean chord Length profile depending on 5T-CNF dose.

The effect of OCC handsheets flocculation with 5T-CNF was also studied in Fig. 10 in which mean chord length (MCL) during the flocculation process at different doses was evaluated. The incorporation of CNF to OCC does not show differences in MCLo before CS due to the small size of CNF without flocculation is not visualized. Unlike when LCR-1-CMF was used, the use of 5T-CNF does not increase the MCLmax by the same amount after CS addition. During the flocculation process with CS, higher doses of 5T-CNF result in lower increases of MCLmax. When the stirring speed increases to 500 rpm, flocs start to break due to high shearing forces and MCL returns to values close to the initial MCLo. Then, when stirring speed was decreased again to 200 rpm, flocs were reflocculated achieving the previous MCLmax value, although far from the MCLo value of OCC handsheets without CNF.

There is little information about how CMF produced by LCR influences the flocculation process. Therefore, floc properties of recycled OCC reinforced with LCR-1-CMF were compared with the study of Merayo *et al*.^50^ that analyze the flocculation behavior of old fluting paper before the corrugation step. They studied two CNF from a never dried refined *Eucalyptus globulus* ECF bleached kraft pulp (TCNF-E) and never-dried corn stalk organosolv pulp from an agricultural waste (TCNF-C). To prepare TCNF-E, they used 5 mmol of NaClO/g of dry pulp in TEMPO-mediated oxidation and six passes of homogenization at 600 bars. The properties were similar to TCNF-E properties shown in Table 3. In order to achieve the same carboxylic groups content in TCNF-C as in TCNF-E, TCNF-C was oxidized with 15 mmol of NaClO/g of dry pulp and the same homogenization passes. TCNF-C properties were similar to TCNF-E with a high nanofibrillation yield and transmittance and a low polymerization degree. The main differences with LCR-1-CMF are in fibrillation size, due to transmittance and nanofibrillation yield of LCR1-CMF being quite low. In addition, polymerization degree in LCR-1-CMF is significantly higher than in chemically treated CNF since mechanical treatments do not break cellulose chains. As expected, the number of carboxyl groups is higher in CNF treated with TEMPO.

**Figure 11:**
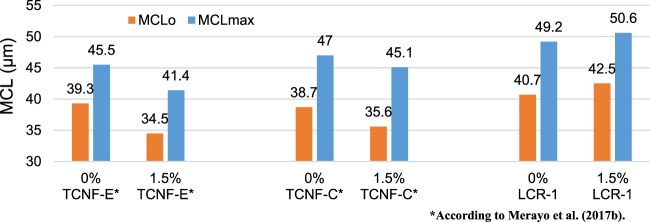
Influence of CMF and CNF treatments in MCL.

Figure 11 shows MCLo and MCLmax of recycled pulp without and with 1.5% CMF/CNF. The results taken from the research of Merayo *et al*.^50^ were obtained from the response surfaces of the study. The addition of CS that produces the variation between MCLo and MCLmax produces an increase in around 20% the MCL of the experiments without significant differences with the type of CMF. However, when 1.5 wt.% CMF/CNF are applied, there are differences in MCLo and MCLmax. When OCC is reinforced with 1.5 wt.% of CNF produced by TEMPO-mediated oxidation, MCLo and MCLmax decrease independent of the use of CS, and the same occurs with 5T-CNF at several dosages. However, these parameters increase when 1.5 wt.% LCR-1-CNF is used. One of the grounds for this difference is fiber size; CNF treated with TEMPO have small fiber size than OCC and for this reason, MCL decrease with their addition. On the other hand, CMF size from LCR is higher and MCL of the mixture increases. Besides, the presence of higher anionic charges during CNF chemical treatments and higher surface area produce a higher consumption of retention systems with a cationic charge and a higher amount of CS is required to maintain flocculation properties.

## Conclusions

CMF treated only with LCR and without additional treatments is a promising alternative to reinforce OCC handsheets instead of other CMF/CNF with chemical treatments and higher production costs. Therefore, high fibrillation is not necessary to improve mechanical paper/cardboard properties. OCC handsheets reinforced with CMF/CNF have improved mechanical properties, and the use of a 6 wt.% LCR-1-CMF and 0.5 wt.% CS as a retention system improves tensile index and bursting strength more than the same dose of chemically treated CNF from NBSK and from other sources. The presence of CMF/CNF reduces drainage but the use of adequate retention systems, such as cationic starch, counteracts this negative effect. In this study, the mechanical treatments to obtain CMF give better results than TEMPO-mediated oxidation pretreatment of NBSK and homogenization. It seems that a high level of refining prevents nanofibrillation by homogenization. Regarding the flocculation process, the higher amount of anionic charges in CNF obtained by chemical treatments produces a higher consumption of cationic retention system. Nevertheless, LCR-1-CNF with low anionic charge does not require the addition of higher retention system doses to improve flocculation.

## Methods

### CMF production by mechanical treatments

The NBSK pulp was highly refined, at the University of British Columbia Pulp and Paper Centre, in a 16-inch low consistency refiner at 1200 rpm and 25 kW of net power to produce CMF. Samples were studied without additional pretreatments (LCR-1-CMF) using 3093 kWh/t of specific energy and with the addition of NaOH pretreatment (LCR-2-CMF) up to a pH of 9, to remove lignin and hemicellulose from cellulose fibers and facilitate fibrillation. In this case, specific energy was 3025 kWh/t and after the refining process, the samples were neutralized with HCl. In order to study the influence of high-pressure homogenization at 600 bar, a laboratory homogenizer PANDA PLUS 2000 manufactured by GEA Niro Soavy (Parma, Italy) was used at the Universidad Complutense de Madrid, Cellulose and Paper Group. LCR-1-CMF and LCR-2-CMF were homogenized using 2 and 4 passes at 600 bars.

### CNF production by chemical pretreatments

To produce CNF from NBSK by TEMPO-mediated oxidation (5T-CNF), 5 mmol of NaClO/g of pulp were used. Once the pulp was oxidized, a cleaning process was performed through filtration steps with tap water. Then, the pulp was homogenized at 600 bars and 3 passes in the PANDA homogenizer.

### CMF/CNF characterization

LCR-CMF and 5T-CNF were characterized by determining consistency, carboxylic groups, nanofibrillation degree, polymerization degree, cationic demand, zeta potential and transmittance according to Balea *et al*.^13^.

Carboxylic groups of oxidized pulp fibers were determined by conductimetric titration before homogenization. A pulp sample containing 0.15 g of dry CMF/CNF was added to 5 mL of 0.01 M NaCl, and deionized water was added to a total volume of 55 mL. pH was adjusted to 2.5–2.8 by adding 0.1 M HCl to protonate carboxylate groups. 0.05 M NaOH was added to the sample in 0.2 mL increments, and the conductivity was recorded after each addition. The number of carboxylic groups was calculated from the curve conductivity vs. NaOH added^51^.

Nanofibrillation degree was determined by centrifugation of 0.1 wt.% of diluted CMF/CNF suspension at 4500 ×*g* for 30 min. The nanofibrillated fraction was isolated in the supernatant, and the yield was calculated as the relation between dry solids in the supernatant and total dry solids^52^.

Polymerization degree (PD) was calculated from the limiting viscosity number of the CMF/CNF suspension, which was determined by the ISO 5351 standard^53^ according to Eq. (1):

1 $${\rm{\eta }}={\rm{K}}\cdot {{\rm{PD}}}^{{\rm{a}}}$$

where **η** is the intrinsic viscosity (mL/g), **K** and **a** have different values according to PD. When PD<950; K = 0.42 mL/g and a = 1, when PD>950; K=2.28 mL/g and a=0.76^13,25^.

The charge of diluted CMF/CNF suspensions was determined by the measurement of both cationic demand and zeta potential. Cationic demand was measured by colloidal titration of 10 mL of 0.1 wt.% diluted CMF suspensions with N-polyDADMAC 0.001 N on a Mütek PCD04 particle charge detector (BTG Instruments GmbH, Herrsching, Germany). In addition, Zeta Potential of CMF/CNF suspensions diluted at the same concentration was measured in a Zeta Potential analyzer Zeta 90 Plus (Brookhaven Instrument Corp., New York, NY, USA).

Transmittance readings of 0.1 wt.% CMF/CNF diluted suspensions were measured in the wavelength range between 800 nm and 400 nm on a Cary 50 Conc UV-visible spectrophotometer using distilled water as reference (Varian Australia PTI LTD, Victoria, Australia).

Finally, CMF were characterized by Scanning Electron Microscopy (SEM) with a JSM 6335 F microscope (JEOL, Tokyo, Japan). Microscopic analyses were carried out in the National Centre of Electronic Microscopy at the Universidad Complutense de Madrid. To prepare the CMF samples for SEM, a drop of diluted suspensions (0.001 and 0.005 wt.%) were dried on a carbon tape at room temperature and covered with a thin layer of Au to avoid superficial charge accumulation. Image J software was used as the image processing program and the diameter range of the CMF suspension was evaluated by measuring the diameter of the fibers located in a diagonal line of different images.

### Pulps and handsheets preparation

Pulps were prepared through disintegration of dry OCC and CMF/CNF in 2000 mL of tap water (1 wt.%) by using a pulp disintegrator from PTI (Vorchdorf, Austria) at 180,000 revolutions, according to ISO 5263–1 standard^54^. The OCC was left to soak 24 hours before disintegration to favor fiber swelling, while CMF (1.5, 3.0, 4.5, 6.0 wt.%) was added just before disintegration. Once the pulp was disintegrated, CS was added as retention agent and stirred for 30 minutes at low speed previous the handsheet formation. Finally, handsheets were prepared with basis weight of 80 g/m^2^ according to ISO 5269–2 standard^55^ by using a Rapid Köthen sheet-former (PTI, Vorchdorf, Austria). Handsheets were conditioned at 25 °C and 50% humidity for at least 24 hours before physical and mechanical characterization.

### Handsheets characterization

Mechanical properties were determined by measuring the bursting strength index (kPa·m^2^/g), Short-span compressive test (SCT) index (N·m/g) and tensile strength index (kN·m/kg). Tensile strength was measured in an MTS Criterion Mode 43 from MTS Systems Corporation (Eden Prairie, MN, USA), following ISO 1924–3 standard^56^. Bursting strength was measured in a Messmer Büchel digital hydraulic board burst tester according to standard ISO 2759^57^ (Veenendaal, Netherlands). To measure the cross-directional short span compressive strength a short span compression tester (Messmer Büchel, Veenendaal, Netherlands) was used according to TAPPI T826 standard^58^. Finally, Bendtsen porosity (μm/Pa·s) was measured with a Bendtsen Porosity Tester n° 8699 from Andersson & Sørensen (Copenhagen, Denmark) according to ISO 5636-3^59^.

### Drainage and retention experiments

As in handsheet preparation, to carry out drainage experiments, OCC and CNF/CMF were disintegrated in the pulper (1.0 wt. %) and the same CMF/CNF doses as for handsheets was prepared. The pulp was then diluted to 0.5 wt.% consistency with tap water. Drainage measurements were made in a Drainage Freeness Retention Tester (MütekTM DFR-05) from BTG Instruments (Herrsching, Germany). Experiments were carried out in duplicate with 500 mL of recycled diluted pulp containing CMF/CNF. Drainage experiments were prepared with and without CS addition. After an initial stirring at 300 rpm and 30 s, CS was added to the pulp if it was required, and mixed for 30 s. Then, the stirring was stopped and the pulp suspension was drained by gravity through a 150 mesh stainless steel wire. A computerized balance recorded the mass of drained water with time (drainage curve). Total solids retention was determined through gravimetric analysis of the drained water at 105 °C and ash retention was determined by incineration at 525 °C^60^.

### Flocculation experiments

The flocculation process is essential to ensure retention of fibers, fines and mineral fillers. In addition, the properties of flocs have a great influence on drainage and the quality of the final product. Flocculation and floc properties of OCC containing CMF/CNF were studied according to the methodology developed by Blanco *et al*.^61^. Decoupling the effects of NC on wet-end and paper properties by using synergies with retention aids, chemical modification, or filler preflocculation is a challenge for the industrial use of CMF/CNF in papermaking^62^. The evolution of flocculation process was monitored in real time by using an M500L focused beam reflectance measurement probe (FBRM) from Mettler Toledo (Columbus, USA) which provides every 5 s a chord length distribution of suspended solids with dimensions between 1 and 1000 µm. In a typical flocculation experiment, the FBRM probe was placed in the suspension stirred at 200 rpm. After 2 minutes of stirring, CS was added. Stirring was kept constant during 3 minutes to observe flocs evolution to study their stability. Then, the stirring speed was increased to 500 rpm over 30 s to break down the flocs in a deflocculation stage, to study the strength under high shearing forces. Finally, the stirring speed was decreased back to 200 rpm to study how flocs reflocculate.
